# An Embedded, Multi-Modal Sensor System for Scalable Robotic and Prosthetic Hand Fingers

**DOI:** 10.3390/s20010101

**Published:** 2019-12-23

**Authors:** Pascal Weiner, Caterina Neef, Yoshihisa Shibata, Yoshihiko Nakamura, Tamim Asfour

**Affiliations:** 1 KIT Department of Informatics, Karlsruhe Institute of Technology, 76137 Karlsruhe, Germany; caterina.neef@th-koeln.de; 2 Department of Mechano-Informatics, University of Tokyo, Tokyo 113-8656, Japan; shibata@ynl.t.u-tokyo.ac.jp (Y.S.); nakamura@ynl.t.u-tokyo.ac.jp (Y.N.)

**Keywords:** sensorised fingers, tactile sensors, parametric model, robotic fingers, prosthetic fingers, hand prostheses, anthropomorphic robotic hands

## Abstract

Grasping and manipulation with anthropomorphic robotic and prosthetic hands presents a scientific challenge regarding mechanical design, sensor system, and control. Apart from the mechanical design of such hands, embedding sensors needed for closed-loop control of grasping tasks remains a hard problem due to limited space and required high level of integration of different components. In this paper we present a scalable design model of artificial fingers, which combines mechanical design and embedded electronics with a sophisticated multi-modal sensor system consisting of sensors for sensing normal and shear force, distance, acceleration, temperature, and joint angles. The design is fully parametric, allowing automated scaling of the fingers to arbitrary dimensions in the human hand spectrum. To this end, the electronic parts are composed of interchangeable modules that facilitate the mechanical scaling of the fingers and are fully enclosed by the mechanical parts of the finger. The resulting design model allows deriving freely scalable and multimodally sensorised fingers for robotic and prosthetic hands. Four physical demonstrators are assembled and tested to evaluate the approach.

## 1. Introduction and Related Work

Humans possess a very sophisticated sense of touch that enables intelligent physical interactions with the environment [1]. If the sense of touch is missing, otherwise trivial manipulation tasks are difficult or impossible to achieve despite visual feedback [2]. Therefore, efforts have been made to replicate the sense of touch in artificial systems such as robotic and prosthetic hands. An overview of different technologies that were explored to create such tactile sensors can be found in [3,4,5,6], a review on the use of tactile information for robotic manipulation is found in [7].

While contact and force sensing play a key role for the control of artificial hands, other modalities contribute to the understanding of the interaction between an artificial hand and its environment. Joint angle sensors can provide information regarding the shape of a grasped object, temperature sensors measure thermal conductivity of an object, accelerometers can detect slip and distance sensors are able to detect the presence of an object before it makes contact with the hand.

Integration of such multi-modal sensor technologies into robotic and prosthetic hands is a complex and challenging task as sensing electronics and electric connections need to be distributed throughout the already complex mechanical structure of an artificial hand and its fingers. An additional challenge is given by the tight space constraints inside fingers and joints. A high degree of integration of the signal conditioning electronics is therefore desirable in order to save space, ensure low noise of the sensor signals and keep the overall system complexity manageable. A low wire count is beneficial as well for reducing system complexity, especially when routing cables through narrow finger joints. Additionally, a simple production and application process eases prototyping and integration into artificial hands.

Prosthetic hands, in particular, need to be scalable to match the size of the able hand of an amputee [8]. However, even for a humanoid robotic hand of any given size, scalability of the fingers is an issue, since the four fingers require different dimensions in order to ensure an anthropomorphic appearance. To ensure the ability to build individually sized hands, the overhead stemming from designing different versions of fingers should be kept to a minimum.

**Figure 1:**
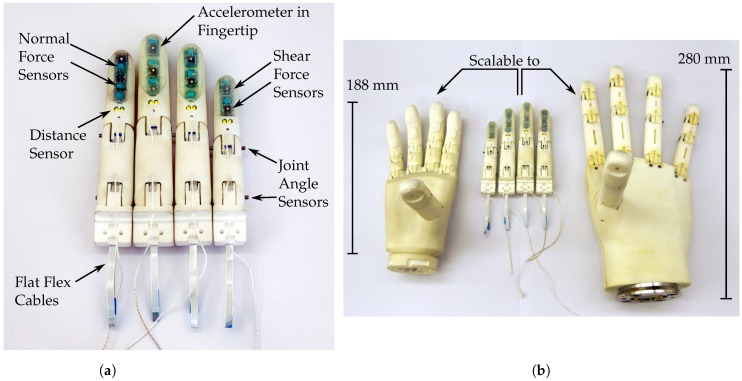
(**a**) The four manufactured demonstrators derived from the scalable model. (**b**) A comparison of the physical demonstrators with our most recent KIT Prosthetic Hand (left) and our robotic KIT ARMAR-6 Hand (right).

In this work, we propose a solution to implement freely scalable fingers based on a parametrised, skeleton-based computer aided design (CAD) model. The fingers are equipped with a multi-modal sensor setup leveraging recent developments in miniaturised commercial sensors for smartphones and consumer electronics. Four physical demonstrators, derived from the presented model, have been assembled and are shown in Figure 1a. We plan to utilise the presented development in differently sized hands as shown in Figure 1b.

There exist a number of approaches that make use of diverse commercially available sensors with integrated signal processing electronics as well as a minimal bus interface to reduce system complexity and size. In many cases a soft material is used to transduce the applied force, protect the electronics from mechanical damage and ensure compliant interaction with the environment.

Tomo et al. [9,10] use commercially available digital three-axis Hall effect sensors together with a magnet embedded into flexible material above the sensor to measure normal and shear forces through the displacement of the magnet. These modular sensors were integrated into the Allegro [11] and the iCub hands and fingers [12,13]. An interesting variation of this sensing method is presented in [14], where a single sensor is used to measure elastic deformation of the finger structure induced by applied forces.

Tenzer et al. [15] use miniature barometric pressure sensors to design tactile normal force sensors. The sensors are completely covered and filled with polyurethane, which acts as the force transmitting medium. When a force is exerted on the soft material, it is transmitted to the sensor and is measured as a change in pressure. These sensors have been integrated into the iHY hand [16]. The design is adapted by Jentoft et al. [17] to allow for a flexible and stretchable array of sensors.

A multi-modal sensor setup is used in [18] to assemble small hexagonal printed circuit boards (PCBs) for the use as artificial skin. Each hexagon houses a microcontroller, an accelerometer, proximity sensors, and temperature sensors. In an extension of the work, force sensing cells are added [19]. A silicone cover protects the skin from damage. Individual cells can communicate with adjacent cells through an event-driven protocol that allows efficient data transfer over a small number of wires as well as offering redundancy in case of failures [20]. The skin is used to cover an entire dual arm manipulation platform [21].

For the humanoid robot iCub, capacitive tactile sensors were developed [22] and integrated into the hand. One plate of the capacitor is formed by a flexible PCB, the other by a deformable conductor. Both are separated by silicone foam. The sensor signal is digitalised using a commercially available capacitance-to-digital converter. In an extension of the work, the production process is optimised in order to make the sensors easier to manufacture and more robust [23]. The capacitive sensing principle is also used for skin spanning larger parts of the iCub robot [24]. Alagi et al. [25] expand on the principle of capacitive tactile sensors by incorporating capacitance-based distance sensing. This sensor is also incorporated into an anthropomorphic hand [26].

In [27], Wörn and Weiss introduce a resistive sensing method using conductive foam and interlaced traces on PCBs. When the foam is compressed onto the traces, resistance between the traces is lowered. Kõiva et al. implement a tactile fingertip based on this measurement method using a PCB lasered onto a three-dimensional structure for the Shadow hand [28]. Recently, this fingertip was extended by a fingernail [29] measuring static forces by combining methods by Tenzer et al. [15] and Tomo et al. [10]. In addition, dynamic events are measured using a three-axis accelerometer. A similar method for force measurement is used in [30] to construct curved tactile fingertips using a flexible PCB and conductive rubber.

Three tactile systems using commercially available distance sensors have recently been introduced [31,32,33]. The sensors are covered by a layer of a translucent elastomer, allowing the sensor to measure distance even through the material. When an object comes into contact with the elastic layer, the layer width is reduced, which can also be measured by the distance sensors. The measured distance can then be translated into a force measurement as the material properties are known. All three systems were evaluated on robotic platforms.

There exist a number of works dedicated to the engineering challenges associated with the design of artificial anthropomorphic fingers. Laurentis et al. [34] present an approach based on a 3D-printed model where all parts of the finger are already assembled during printing, thus reducing production time. Only the sensors have to be added after the printing process. Liu et al. present a prosthetic hand with fingers able to withstand deformations and impacts, while also embedding a three-axis force sensor [35]. The fingers are made of individual segments connected by ligaments and rolling contact joints. The fingertips are equipped with three-axis force sensors based on an optical measurement principle [36]. Cheng et al. [37] incorporate a tactile sensor fingertip and joint angle encoders in an anthropomorphic prosthetic finger fabricated from metal. The finger is actuated by a motor directly attached to the finger with a rigid transmission mechanism, all encapsulated inside of the finger. The design of a prosthetic finger with integrated force sensing is presented by Imbinto et al. [38]. The included sensor is based on the work by Jentoft et al. [17]. The finger includes a motor in the proximal phalanx as well as a non-back driveable mechanism that locks the finger while grasping.

The multimodal BioTac tactile fingertip (SynTouch Inc., Montrose, CA, USA) incorporates 19 electrodes embedded into a fluid-filled soft fingertip.When pressure is applied to the fingertip, the distribution of the liquid inside the finger changes and the resistance between the different electrodes changes with it. A hydro-acoustic pressure sensor measures high frequency vibrations through the liquid and additional temperature sensors in combination with heating elements enable temperature flux measurements [39]. The rich set of information generated by the multimodal sensor setup used in the BioTac sensor is well suited for feature extraction and classification based on machine learning approaches [40].

With the development of sensorised fingers we set out to provide a rich multi-modal sensory feedback while addressing the challenge of tight system integration, aiming at a practical and useable yet versatile solution. We intend to use these fingers in both our prosthetic hand developments [41] as well as the hands for our humanoid robot ARMAR-6 [42]. As with our first version [43], we make use of commercial components and proven sensing methods in order to limit system complexity while introducing scalability, improving robustness, the anthropomorphic design, and extending the sensor suit. The developed sensor system allows sensing of normal and shear forces, distance, vibration, joint angles, as well as temperature. The finger’s mechanics and its electronics are freely scalable to the dimensions of any human finger, which to the authors’ knowledge, is the first solution to address this problem in literature. We consider the integration of a rich sensor suit into a scalable and robust robotic finger to be the major contribution of this work.

In the remainder of this work we give a detailed overview over the mechanical and electrical design of the fingers. We describe how the central requirement of scalability influences the design and how the multi-modal sensor system is integrated into the finger. Finally, extensive tests are conducted to characterise the different sensor modalities individually and as a system to show the validity of our approach.

## 2. Design of Anthropomorphic Robotic Fingers

With our development of robotic fingers with a multi-modal sensor system we strive to enhance grasping and manipulation skills of both prosthetic and robotic hands as well as to enable haptic feedback for prosthetic applications. When used as robotic fingers, the sensor system should provide sufficient information for closed-loop control throughout all phases of the grasping process, namely approach/pregrasp, contact with the object, lift, placing and breaking contact. In a prosthetic setting the fingers should enable haptic feedback to the user. The information provided by the sensor system can also be used for semi-autonomous control schemes where the prosthesis automates part of the grasping process. This relieves the user from the high cognitive burden of manually controlling a prosthetic device.

As especially prosthetic hands should match the dimensions of the human hand, it is important to be able to scale the dimensions of the model and manufacture new fingers easily. This free scalability is particularly challenging for the embedded electronics. Application of such fingers in robotic, as well as prosthetic, settings also means that the requirements regarding the sensor setup vary in terms of sensor position, quantity and type of sensors. The mechanical design and electronics must be flexible enough to allow for such reconfiguration.

Different sensor modalities have been proven to be useful in the robotic and prosthetic domain. Apart from normal and shear force sensing, accelerometers enabling slip detection, as well as distance sensors have been used. For both robotic and prosthetic hands, under-actuation is an important principle for building lightweight hands with simplified control strategies, where multiple joints are coupled and actuated jointly. Such under-actuated hands rely heavily on exploiting interaction and contact with the objects and environment to perform successful grasping. For such hands, contact and force information is essential and joint angle encoders are needed for estimating the current kinematic state of the hand.

Our approach to implementing a multi-modal sensor system into an anthropomorphic finger combines a completely parametrised CAD-model with a modular electronic system consisting of commercially available sensors and standard PCBs. For force sensing we combine and extend methods described by Tenzer et al. [15] and Tomo et al. [10]. Additionally, we incorporate distance, vibration and joint angle sensing into the system. Mechanical parts are realised using 3D-printing. In the remainder of this section we describe the interplay of mechanical scalability and electrical modularity and how these two central concepts are implemented in detail.

### 2.1. Finger Skeleton

**Figure 2:**
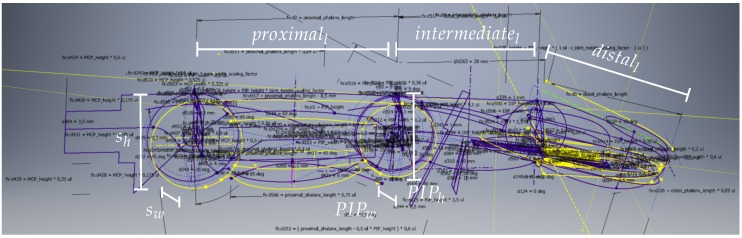
Skeleton sketches used for the individual phalanges. The seven high-level parameters are used in these sketches to derive all dependent dimensions of the finger.

For the mechanical structure of the fingers we created a single scalable CAD-model of the finger. It is based on a skeleton that contains all sketches for all important features of the finger, as can be seen in Figure 2. Based on this skeleton, the three individual parts for knuckle, proximal and distal phalanx are derived by referencing the sketches in the skeleton model. The skeleton can be parametrised using seven high-level parameters that can be set individually and independently of each other. These parameters define the width and height of the proximal interphalangeal (PIP) joint ($PIP_{w}$ and $PIP_{h}$) as well as the lengths of the proximal, intermediate and distal phalanx ($proximal_{l},\;intermediate_{l}$ and $distal_{l}$). Two additional parameters $s_{w}$ and $s_{h}$ represent scaling factors that are used to determine the width and height of the distal interphalangeal (DIP) and metacarpophalangeal (MCP) joints as follows:

MCPw=PIPw·sw  DIPw=PIPw·(1−(sw−1))MCPh=PIPh·sh  DIPh=PIPh·(1−(sh−1))

The scaling factors $s_{w}$ and $s_{h}$ are calculated as the average ratios of joint height/width as measured by Vergara et al. [44]. If desired, the height/width of all joints can also be changed individually and independently by replacing the above calculations for MCP and DIP in the root sketch with independent scalar values.

The seven parameters ($PIP_{w},\;PIP_{h},\;proximal_{l},\;intermediate_{l},\;distal_{l},\;s_{w},\;s_{h}$) define lengths and radii in a root sketch, together with basic definitions of useful axes and planes. This root sketch is referenced by further sketches in the model that define further features like loft guides or faces later used for extrusions. The model makes use of splines which allow for round and smooth shapes. To keep the number of spline parameters small, we define the whole shape of the finger with as few splines as possible and use a small number of via points for each spline. Once the whole skeleton is defined as a set of sketches, the three individual parts of the fingers are designed by deriving relevant sketches from the skeleton and building the geometry based on these sketches.

In addition to the seven high-level parameters, the model contains 257 other parameters, which are either derived from the high-level parameters or are constant like fittings, so that a change in high-level parameter values results in a change of the dimensions of the entire finger.

**Figure 3:**
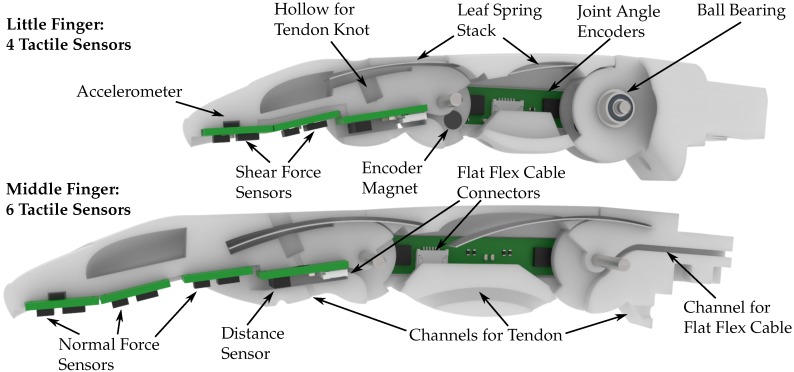
Section view of the little and middle finger with the model scaled to the 50^th^ percentile female dimensions. The section view of the little finger shows the joints of the finger while the deeper cut into the middle finger shows the paths for both the flat flex cables (FFCs) and the tendon.

The parameters can, for example, be set to match the measured dimensions of the able hand of an amputee. We successfully tested the model using the 5^th^ percentile female hand dimensions as well as the 95^th^ percentile male hand dimensions. Figure 3 shows two specimens of the developed CAD-model scaled to the dimensions of the little and middle finger sized according to a median female hand.

**Figure 4:**
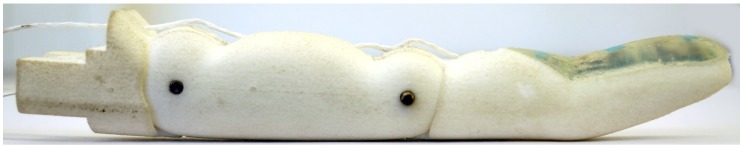
Profile of the ring finger showing a curved design for the individual phalanges.

In addition to human sizing, special attention was given to the anthropomorphic shape of the fingers which is an important factor especially for the acceptance in prosthetic applications [45]. In Figure 4, the profile of a physical demonstrator is shown.

We consciously made the decision to fuse the distal and intermediate phalanx into one part. Each finger hence has two joints, the MCP joint and the PIP joint. Omitting the DIP joint reduces the complexity of the assembly and allows for sufficient space in the distal part to house the sensor system. This is a common design choice for both commercially available prostheses [46] as well research prostheses and anthropomorphic robotic hands [47].

### 2.2. Physical Demonstrators

The physical demonstrators in this work were sized according to the 50^th^ percentile female dimensions as described by the German standard specification (DIN 33402-2) in finger length and additional dimensions as identified by Vergara et al. [44]. The dimensions for all fingers and segments are shown in Table 1. As the range for prosthetic and robotic hand sizes varies greatly, we have chosen dimensions at the smaller end of the range to ensure that the model and electronics can be used in even small hands and can consequently also be easily modified and extended for larger hands.

The individual components for the fingers are manufactured from polyamide (PA 2200) using 3D-printing methods (selective laser sintering) to enable individualised sizing as needed. Through the use of additive manufacturing for mechanical parts, commercially available sensors and standard manufacturing techniques for the PCBs the price for an individual finger can be kept lower than 70.

### 2.3. Joint Structure and Actuation

Each finger consists of three individual parts, the distal/intermediate phalanx, the proximal phalanx and the knuckle, as well as two joints, the metacarpophalangeal (MCP) joint and the proximal interphalangeal (PIP) joint, that connect the phalanges. Each joint is supported by two miniature metal ball bearings to reduce friction to a minimum. The joints are actuated in flexion direction by a tendon that is located on the palmar side of the fingers. Actuation via tendons was chosen as it does not require levers inside of the finger, hence leaving necessary space for the electronics and cables.

Each joint is extended by a stack of leaf springs, as opposed to the torsion springs, that are used in the previous version of the KIT prosthetic hand [41], in order to free up space in the joints for sensors and their cables. The leaf springs are installed completely inside each finger and are not visible from the outside. The pockets for holding the leaf springs are slightly curved to minimise friction and simultaneously create pretension in the springs. One end of the leaf springs is glued into the phalanx, the other slides in and out of the pocket. In order to decrease the friction between the finger material and leaf springs, we created a hollow space inside the finger. Both the distal/intermediate as well as the proximal phalanx are hollow for the most part to allow the springs to extend without friction and in order to save weight. We used spring steel with 0.1 mm thickness as the leaf spring material. Each joint is equipped with a stack of three leaf springs to reach a sufficiently high torque.

### 2.4. Embedded Sensor System Overview

As shown in Figure 1 and Figure 3, a customizable number of sensors can be integrated into a finger, depending on the finger pad area available for the integration of tactile sensors. The middle, and thus largest, finger used in this work, shown in the bottom of Figure 3, contains a total number of ten sensors, which include two joint angle encoders, a distance sensor, three normal force sensors, three shear force sensors and one accelerometer. The sensor PCBs can be connected to a controller using FFCs via connectors on the joint angle encoder and distance sensor PCBs (see Figure 3), while the tactile sensor PCBs are connected through magnet wires.

### 2.5. Sensor Placement Experiments

**Figure 5:**
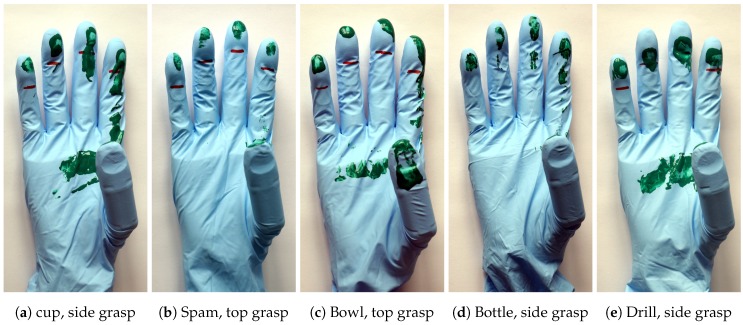
Examples of results from the finger contact surface experiment.

In order to find the optimal configuration and placement positions of the tactile sensors in each finger, we conducted tests to determine which surfaces of each finger are in contact when grasping different objects. These experiments were carried out prior to the definition of the tactile sensor layout and their results were used to define the area on the finger that should be covered with tactile sensors. We used the 50^th^ percentile female version of the KIT Prosthetic Hand [41], as well as five objects (banana, baseball, bowl, drill, spam) from the YCB object set [48] and two objects (cola, green cup) from the KIT object set [49], in order to have a variation of shapes and sizes. The outside surface of each object was painted green, after which the object was immediately grasped using either a top or a side grasp (approaching the object from the top or the side, respectively). From the experiments it is evident that the finger area that is in contact with most objects is the finger pad, especially that of the index finger. Examples of the experiment results can be seen in Figure 5. Thus, contact forces and slip can be measured most accurately by placing sensors in these areas.

### 2.6. Sensor System Configuration

**Figure 6:**
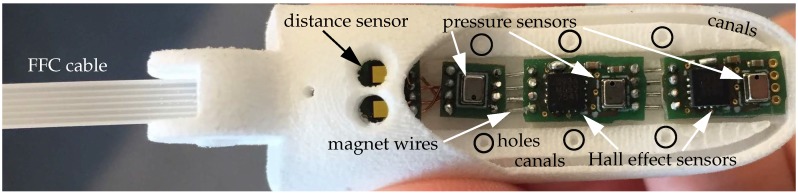
The individual tactile sensor printed circuit boards (PCBs), containing normal and shear force sensors and an accelerometer, as well as the distance sensor and design details are shown.

We use a combination of three types of sensors to acquire a wide range of tactile information in the available finger pad area, shown in Figure 6. The normal force sensors (NPA201, Amphenol, Wallingford, USA) are based on ideas presented by Tenzer et al. [15]. In contrast to the design used by Tenzer et al., they are constructed by leaving a small pressure chamber in the silicone above the barometric pressure sensor as explained in [43]. When a force is exerted on the silicone in vicinity to the sensor, this force compresses the pressure chamber which in turn is measured as an increase in pressure by the sensor. These sensors will be called barometer-based sensors in the rest of this publication. While estimating forces only in one direction, they are very sensitive and offer a high resolution, as shown in Section 3.1. The shear force sensors (MLX90393, Melexis, Ypres, Belgium) are based on work presented by Tomo et al. [9,10]. They can be used to estimate both normal and shear forces and offer a larger measurement range than the normal force sensors. These sensors will be called Hall effect-based sensors in the rest of this publication. The normal force estimation is hence performed using different measurement principles for each sensor, enabling a more accurate overall measurement and event detection through sensor fusion.

An accelerometer (BMA456, Bosch Sensortec, Reutlingen, Germany) with a sample rate of 1.6 kHz is mounted at the back of the most distal sensor PCB. It can be used to detect slip of grasped objects as it is, unlike the barometer- and Hall effect-based sensors, able to achieve the necessary sampling rates for this task.

To gain additional information even before contact is made with an object, we implemented a distance sensor into the finger, shown in Figure 6. The sensor is a state of the art time of flight (ToF) device that is able to measure the distance of objects independent of their reflectance (VL53L1X, STMicroelectronics, Geneva, Switzerland). The distance information acquired with this sensor could for example be used to control an artificial hand to form a pre-grasp when approaching the object, as it can determine the distance between the finger and the object which in turn can then be used to adjust the pre-grasp accordingly.

All sensors in the sensor system are commercially available sensors that integrate all signal conditioning and digitalisation circuits. All sensors also include a temperature sensing element that can be sampled together with the main sensor signal. This way no additional electronics are needed for signal processing. All sensors, except for the accelerometer, are sampled with 50 Hz, while the accelerometer is sampled at 1.6 kHz. All sensors communicate using the two-wire I_2_C communication bus. Together with the supply voltage lines, only four wires are needed to operate and read out all sensors. As there are no commercially available flat flex cables and connectors with four terminals, the smallest available configuration with six terminals is chosen to connect the sensor system to a central processor, typically inside of the palm of the robotic hand.

### 2.7. Manufacturing Process

The manufacturing process of the tactile sensors is described in the following. First the tactile sensor PCBs for each finger are glued into place. We then use the same methodology described in our previous work [43] to cast the barometer- and Hall effect-based sensors in silicone rubber.

For the Hall effect-based sensors a thin pad of silicone with Shore hardness A (ShA) 13 is glued to the top of the sensor using silicone glue. A magnet is placed on the centre above the sensor and a drop of silicone is used to fix the magnet to the silicone pad.

**Figure 7:**
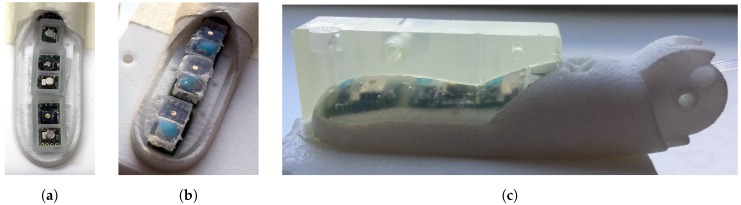
(**a**) The moulds for the silicone rubber casting process are placed directly into the finger. (**b**) The individual sensors have been cast in rubber. The next step is to cast the remaining finger pad area in rubber. (**c**) The result of the casting process of the finger tip and one half of the used mould is shown. A silicone canal is used to inject silicone rubber into the moulds.

For the barometer-based sensors a small mould is placed around the sensor. A small magnet is placed directly above the opening in the casing of the barometric pressure sensor and the mould is filled to the top of the magnet with silicone (ShA 45). Since the housing of the pressure sensor is magnetic, the magnet is tightly held in place. As soon as the silicone is hardened, the magnet is removed. The resulting hole forms the walls of the pressure chamber above the sensor. The hole is then covered with a thin sheet of silicone (ShA 22) and fixated by a drop of silicone. Both moulds and placeholder magnets are shown in Figure 7a. The 3D printed moulds (as shown in Figure 7a) can be placed directly onto the PCBs glued into the finger. The individual sensors are cast in rubber, as shown in Figure 7a, the result of which can be seen in Figure 7b.

The entire area of the finger pad is then cast in an additional layer of silicone rubber (ShA 13), shown in Figure 7c. In order to ensure sufficient stability of the silicone layer, holes and canals with undercuts, as shown in Figure 6, are integrated into the finger tip. This design allows for increased adhesion of the silicone to the 3D printed material. Compared to the previous work, a larger part of the fingertip is cast in silicone rubber to enable a configuration with a larger number of sensors. This also contributes to an improved grasping behaviour as the silicone rubber is more elastic and has a higher friction coefficient compared to the 3D printed material [50].

### 2.8. Joint Angle Measurement

Additionally required for the forming of pre-grasps are joint angle encoders that determine the rotation angle of each joint and can be used to control and adjust the finger flexion. One possibility to measure joint rotation angles is to place a diametrically polarised magnet directly on the joint axis that rotates with the joint. A Hall effect sensor that is placed directly above the magnet is then able to measure the change in magnetic field strength induced by the rotation of the magnet. As the Hall effect sensor outputs the magnetic field strength in *x*, *y* and *z* direction, these values can be used to derive the rotation angle of each joint.

To calculate the rotation angle $\alpha_{z}$ around the z axis, the following equation is used, where $x_{Mag}$ and $y_{Mag}$ are the magnetic field strengths in x and y direction, respectively:

(1) $$\alpha_{z}=\arctan2\left(y_{Mag},x_{Mag}\right)\;\cdot \;\frac{180^{\circ }}{\pi }$$

**Figure 8:**
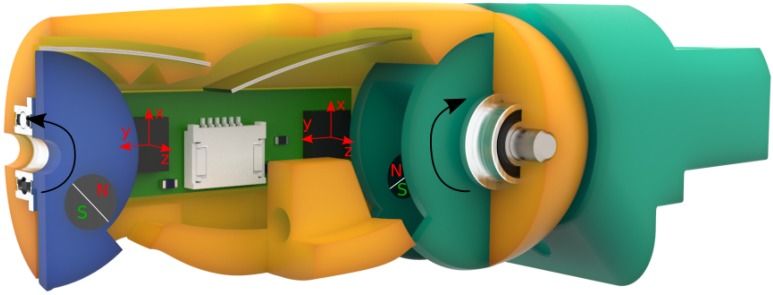
Section view of the little finger, with the distal/intermediate phalanx in blue, the proximal phalanx in orange and the knuckle in green, showing the joint angle encoders and magnets used to determine the joint rotational angles.

In this work, however, we perform this measurement off-axis both for the magnet and the sensor (MLX90393, Melexis), due to space constraints in the joints, which is shown in Figure 8. The magnets are glued into the distal/intermediate phalanx (blue) and the knuckle (green), and rotate around their respective joints when these are rotated. At 45 degrees rotation the magnets are positioned directly above the sensors, so that they have the same distance between each other at 0 and 90 degrees, which corresponds to the minimum and maximum angle, respectively, of all joints. The above stated Equation (1) can nevertheless be used to provide an approximation of the joint rotation angle using this off-axis measurement. However, when the placement of the magnet above the sensor changes in *x* or *y* direction, the magnetic field strength and therefore sensor output of the Hall effect sensor changes. Therefore, an experiment is necessary to determine the correlation between the sensor output and the actual rotation angle, which is described in Section 3.3.

As the PCB for the joint angle encoders contains just two sensors and a connector, it is fairly easy to adjust the distance between the two sensors for different finger sizes directly in the PCB layout. We hence produced PCBs in three different sizes for the little finger, the middle finger, and the other two fingers which can accommodate the same PCB size due to their similar dimension.

## 3. Experimental Results

**Figure 9:**
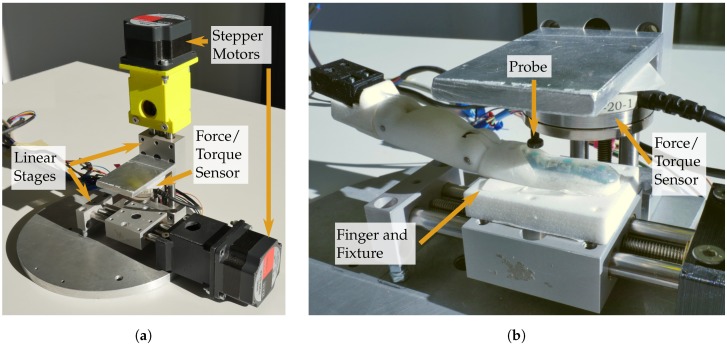
Linear table for normal force, shear force and spatial mapping experiments; (**a**) depicts an overview of the linear table; (**b**) shows a close up of the f/t sensor and finger attached to the axes

A series of experiments were conducted on the physical demonstrators to assess the performance of the sensors individually and as a coherent system. For the experiments regarding normal and shear forces, as well as a spatial mapping, a two-axis linear table was used as depicted in Figure 9. Each axis was a precision linear stage (PT4808, MM Engineering GmbH, Brackenheim, Germany) with 0.5 mm displacement per turn attached to a stepper motor with 200 steps per turn. A force/torque sensor (Mini 40, ATI Industrial Automation, Apex, NC, USA) was mounted on one axis. The sensor could be equipped with a cylindrical probe with a diameter of 5.3 mm, which was small enough to allow applying loads to individual sensors. A sensorised finger could be attached to the other axis, enabling the probe to apply normal forces to different parts of the finger along one axis, as well as shear forces when the finger was moved while normal forces were applied.

The communication with the sensors during the experiments was implemented on the embedded system also used in the KIT prosthetic hand [41]. This embedded system was based on a microcontroller (STM32H7 series, STMicroelectronics, Geneva, Switzerland) and provides four I^2^C ports.

In general, we tried to incorporate sensors from different fingers into the experiments to examine if they exhibited similar characteristics. The following experiments for the tactile sensors were intended to determine that the methods described by Tomo et al. [9,10], Tenzer et al. [15] and Weiner et al. [43] could be successfully adapted despite differences in design like smaller magnets and the curved shape of the finger. A thorough characterisation of the tactile sensor technologies used in this work can be found in the works above. The experiments primarily give an overview of the quality, correlation and coherence of the signals generated by the different sensor modalities.

**Figure 10:**
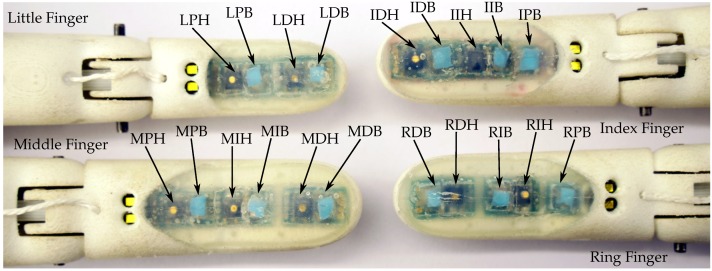
Identifiers for all sensors on all physical demonstrators. The identifiers are named after the first letter of the finger name, their position inside the finger and the sensor type.

To be able to identify the individual tactile sensors for the experiments we adopted the following naming scheme: the sensor names started with the beginning letter of the finger they were included in—I for index finger, M for middle finger, R for ring finger, and L for little finger. The second letter denoted the position inside the finger—D for distal, I for intermediate and P for proximal. Note that the little finger did not have intermediate sensors. The third letter distinguishes the type of sensor—H for Hall effect-based sensors and B for barometer-based sensors. The Hall effect-based sensor at the tip of the index finger would hence be IDH. An overview over the positions of all tactile sensors and their corresponding designators in all physical demonstrators can be seen in Figure 10.

### 3.1. Normal Force Sensor Characterization

**Figure 11:**
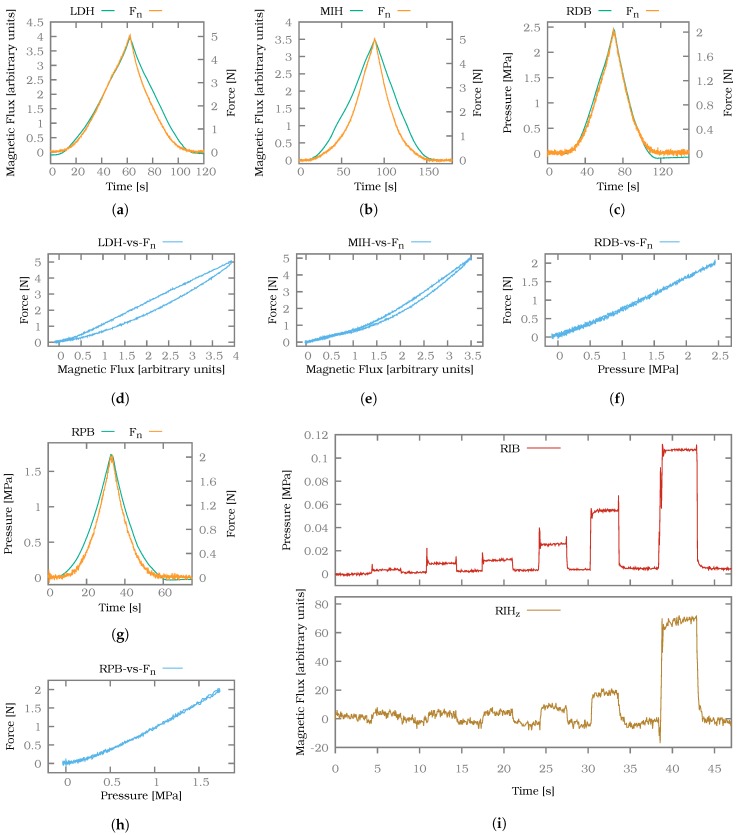
(**a**,**b**) Normal force measurements for Hall effect-based sensors. (**d**,**e**) Corresponding hysteresis plots. (**c**,**g**) Normal force measurements for the barometer-based normal force sensors. (**f**,**h**) Corresponding hysteresis plots for the barometer-based sensors. (**i**) Weights distributed on a Hall effect- and barometer-based sensor.

Two types of normal force sensors were built into the fingers. The barometer-based normal force sensors were able to resolve small forces but also saturate at comparatively low forces. The Hall effect-based sensors did not offer the same level of resolution but were able to measure magnitudes higher forces before saturation sets in. For the normal force experiments we used the aforementioned linear table (see Figure 9) to allow applying and measuring well-defined forces. In Figure 11 two measurements for Hall effect-based sensors (a,b) and two measurements for barometer-based sensors (c,g) are presented in green. The ground truth measurement of the force/torque sensor is plotted in orange (labelled $F_{n}$). These two measurements together are combined in the hysteresis plots (d–f,h) corresponding to the four sensor measurements (a–c,g).

To show the difference in resolution we carried out an additional experiment where a small metal plate was placed on the adjacent sensors RIB and RIH on the ring finger. The plate distributes the load of any weight placed in its centre evenly on the two sensors. For the experiment consecutive weights with an increasing mass of 0.4 g, 0.85 g, 1.1 g, 2.2 g, 4.65 g and 10.75 g were placed on the plate. The resulting sensor readings can be seen in Figure 11i.

Both types of sensors were able to track the applied normal forces. Differences were visible in the hysteresis behaviour as the barometer-based normal force sensors RDB and RPB showed a more linear correspondence between their signals and the normal forces measured by the force/torque sensor. Furthermore, the hysteresis was directed in different directions for both sensor types while unloading the sensor. While the Hall effect-based sensors LDH and MIH showed a notable lag when returning to the unloaded state compared to the ground truth, the barometer-based sensors overshot the unloaded state.

In terms of sensitivity the barometer-based sensors had a clear advantage over the Hall effect-based sensor as can be seen in Figure 11i. The barometer-based sensor RIB showed a discernible response even to the smallest weight of 0.4 g, whereas the noise in the signal of the Hall effect-based sensor RIH only allowed the detection of the fourth 2.2 g weight with sufficient confidence. The barometer-based sensors saturated by design at 2.6 MPa which was only slightly above the maximum sensor readings observed during the above experiments at 2 N. The Hall effect-based sensors on the other hand showed a clear signal at forces up to 5 N.

Overall the barometer-based sensors offered a good performance for low forces coupled with a comparatively low hysteresis. The Hall effect-based sensors offered a far wider sensing range at the expense of a more nonlinear behaviour and a stronger hysteresis effect, which could arguably also be caused by the applied forces being higher.

### 3.2. Shear Force Sensor Characterization

**Figure 12:**
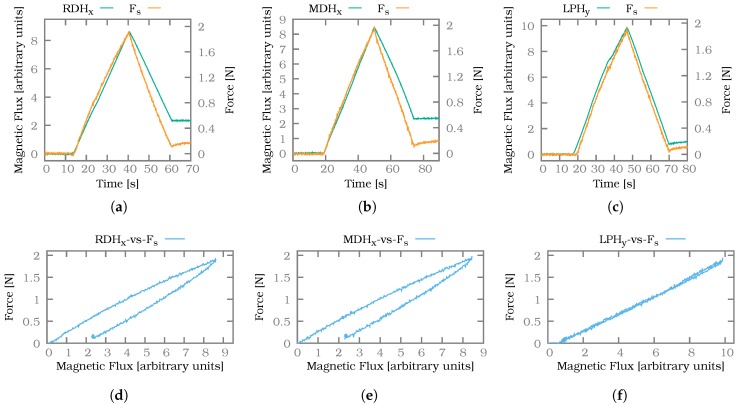
Exemplary shear force measurements for sensor signals RDH_x_ (**a**), MDH_x_ (**b**) and LPH_y_ (**c**) as well as the corresponding hysteresis plots (**d**–**f**).

To reliably allow applying shear forces, the force sensor without a probe was used to first apply a normal force of 5 N to the fingertip. The larger sensor surface compared to the probe then allowed to evenly shear the soft silicone material whereas a small probe would only cause a local and undefined distortion. As soon as the normal force threshold was reached, an increasing shear force was applied by the second axis of the linear table up to a limit of 2 N. After the limit was reached the shear force was lowered again until it reached a value close to zero. The direction of the exerted shear forces was chosen to correspond to one of the two measurement axes of the shear force sensors in the fingers. For the experiments, shear force sensors in the ring finger (x-axis), middle finger (x-axis) and little finger (y-axis) were chosen. The resulting measurements can be seen in Figure 12. For each measurement the shear force sensor signal, as well as force/torque sensor values, are plotted in diagrams (a–c) and corresponding hysteresis plots are provided in (d,e).

In general, the shear force sensors are able to correctly track the direction and rate of change of the applied shear forces. The amplitude of the signal is similar for all sensors, although not identical. Due to the anthropomorphic shape of the finger the silicone is not evenly distributed onto the sensors but follows the curved shape of the human finger. Hence different sensors are covered by silicone of different heights as shown in Figure 4 and the amount of transduced pressure changes accordingly. In addition, the force/torque sensor is in almost all cases not perfectly aligned with the sensor plane since the PCBs for the sensors are mounted at a slight angle.

From the three hysteresis plots a significant hysteresis is noticeable for the sensors RDH_y_ and MDH_x_. This is also evident at the end of the plots (a) and (b) as the signal of the shear force sensor remains notably higher than that of the force/torque sensor. For the shear force sensor LPH_y_ in the little finger the hysteresis is much less noticeable. As the little finger is the smallest, the silicone layer on top of the sensor, as well as the overall amount of silicone, is smaller than for the middle and ring finger. Hence the effect of hysteresis should also be reduced for this finger. During the experiments we found no sign of crosstalk between the sensors, meaning the magnet on one Hall-effect-based sensor did not affect the other Hall effect-based sensors. There was also no noticeable crosstalk between the Hall effect-based tactile sensors and the joint angle encoders.

It can be concluded from the above observations that the shear force sensor signals are able to track direction and dynamic of shear forces well. The sensors exhibit notable hysteresis for more static forces. The shape of the finger does not seem to influence the sensor performance too much.

### 3.3. Joint Angle Sensor Characterization

**Figure 13:**
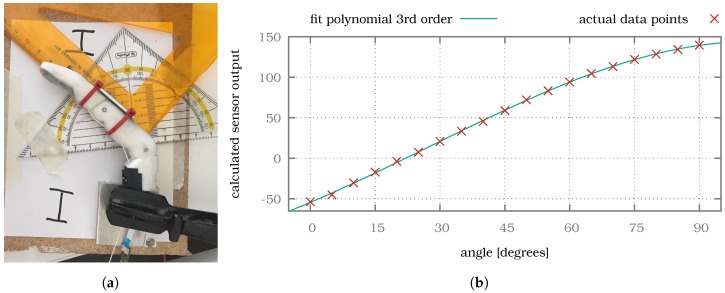
(**a**) The PIP joint is fixed and the MCP joint is incrementally rotated by 5° while the sensor output data are acquired, to determine the correlation between sensor output and actual rotation angle. (**b**) The resulting data points and 3rd order polynomial fit.

As mentioned, due to space constraints the measurement of the joint rotation angles is performed off-axis in this work (see Section 2.4). Therefore, an experiment, shown in Figure 13a for the MCP joint of the index finger, was necessary to determine the correlation between the calculated sensor output $\alpha_{z}$ (using Equation (1), based on the magnetic field strengths $x_{Mag}$ and $y_{Mag}$ in *x* and *y* direction) and the actual rotation angle of the joint. To determine this correlation, we moved each joint of each finger incrementally in steps of 5 ${}^{\circ }$, starting at 0 ${}^{\circ }$ and ending at 90 ${}^{\circ }$, which corresponds to the minimum and maximum rotation angle of each joint, respectively. To ensure that only the correct joint was rotated, we fixed the other joint during the measurements. At each step the sensor output ($\alpha_{z}$) and rotation angle were recorded, after which the joint was bent five degrees further. The resulting correlation between rotation angle and sensor output was then used to obtain a 3^rd^ order polynomial fit for each joint, shown for the MCP joint of the index finger in Figure 13b. This fit can be used for the real time control of the finger to directly calculate the rotation angle of each joint during the data processing step.

As the rotational orientation of the diametrally polarised magnet can not be exactly controlled during assembly, this polynomial fit needs to be experimentally determined for each joint individually if accurate joint angle measurements are needed. The position of the PCBs with the Hall effect sensors inside the proximal phalanx can also vary slightly. Alternatively, the curve can be linearly interpolated using the lowest and highest measured value for increased calibration speed at the cost of angular resolution.

In addition to calibration, we investigated the influence of crosstalk between the magnet of one joint and the Hall effect sensor of the other joint. For this experiment the distal joint was fixated and the proximal joint actuated across the full range while recording the values of the distal sensor. For the little finger, with a minimal distance of 16.7 mm between distal Hall sensor and proximal magnet, we measured a maximum of 1.1 ${}^{\circ }$ of crosstalk. The other fingers did not show significant crosstalk as the distances between sensor and magnet are larger ( 23.3 mm for index/ring finger and 27.3 mm for the middle finger).

### 3.4. Object Grasping and Slip Detection

To evaluate the performance of the multimodal sensor system, we devised a grasping experiment where an object is grasped, held and released using two sensorised fingers. During the holding phase slip is induced. For this experiment the little and ring finger are fixed in direct opposition to each other, meaning both sensor surfaces are roughly facing each other. The tendon of the little finger can be actuated manually so that an object can be grasped in a pinch grasp configuration.

**Figure 14:**
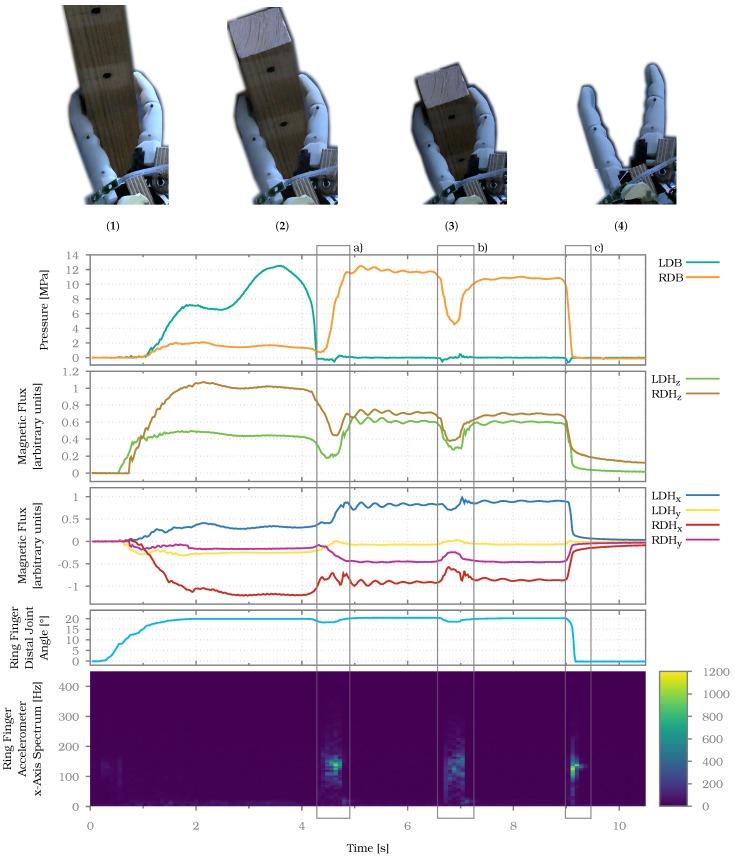
Sensor signals recorded while grasping a block with two fingers in a pinch grasp configuration, letting the object slide twice and releasing the grasp. The top row images (1)–(4) show the static states between the slip events (a)–(c).

Using this setup, a wooden block of 4×4×20 cm and a mass of 215 g is grasped firmly. The holding force is then lowered until slip occurs, after which the grasp is quickly fastened again twice. Afterwards, the grasp is released. All normal-force, shear-force, accelerometer and joint angle sensors for both fingers are recorded simultaneously. Figure 14 shows the signals of the different sensors throughout the experiment. For clarity only changing sensor values are plotted. To make the characteristic frequencies generated by incipient and gross slip visible, the accelerometer values are transformed using a short-time Fourier transformation (STFT).

At the beginning of the experiment the wooden block is grasped just above the centre of mass, as can be seen in Figure 14(1). The distal barometer-based sensor of the little finger LDB and the distal Hall effect-based sensors LDH_z_ and RDH_z_ are loaded. This means that the point of contact on the little finger is located between the LDB and LDH sensors, whereas the contact point on the ring finger is close to the RDH sensor. Both shear force components LDH_x_ and RDH_x_ show a signal proportional to how near they are to the contact point, according to their normal force component. Since the shear force sensor in the ring finger is rotated by 180 ${}^{\circ }$, its values are negative, whereas the values of the shear force sensor in the little finger are positive.

After around 5 seconds the first slip event occurs, marked by box a). Just prior to the event, grip strength is reduced as indicated by all sensors LDB, LDH_z_ and RDH_z_. The reduction of grip strength also results in a slight reduction of the joint angle in the distal joint of the ring finger (fourth plot). The gross slip is detected by the accelerometer y-axis, as can be seen in the STFT of the signal at box a) (fifth plot). As soon as the slip occurs, the grip is manually strengthened again. During the slip event the contact point of the block on the fingers changes, as can be seen when comparing Figure 14(1) and Figure 14(2). On the ring finger it moves between the sensors RDB and RDH, whereas on the little finger it moves away from LDB towards LDH. Hence the normal force sensor RDB gets loaded while RDH_z_ gets partly released. The opposite is true for the little finger. The slip event also induces a small pendulum motion on the wooden block around the two contact points which can be seen in the small waves in all loaded sensors after the first slip event.

The second slip event occurs at around 7 s and is again induced by reducing the grip force, as can be seen in the signals of LDH_z_, RDB and RDH_z_. The joint angle also changes slightly as the grip is released. Again, the slip itself is clearly visible in the signal of the accelerometer in the ring finger. After the slip event the block is grasped near the top, as can be seen in Figure 14(3). At around 10 s the grip is released, causing a very short but intensive slip event. The fingers are fully opened again, as can be seen in Figure 14(4), and is also visible in the joint angle measurement. As can be seen in the last two seconds of the plot, the shear force sensors LDH and RDH exhibit hysteresis after unloading, whereas the normal force sensor RDB returns to zero immediately. The same holds true for the normal force sensor LDB at the time it is unloaded.

The experiment shows that distinct events during grasping, such as making or breaking contact, as well as gross slip, can be detected by not only one single sensor modality but multiple different modalities. This allows for the fusion of sensor data from different modalities in order to gain more confidence for the detection of events during grasping.

### 3.5. Spatial Resolution and Sensitivity

**Figure 15:**
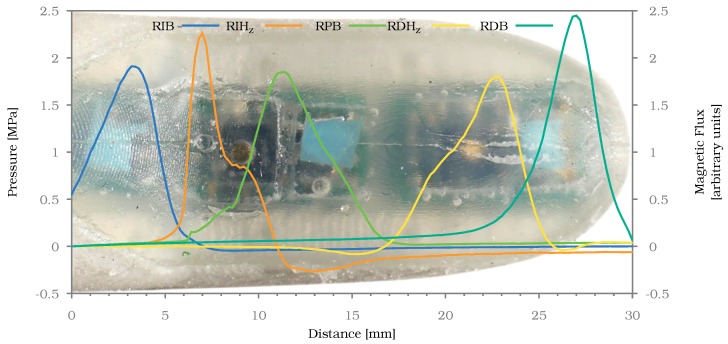
Normal force signals for sensors of the ring finger while probed with 2 N of normal force along the axis from the proximal end of the fingertip to the distal end. The background image shows the approximate position of the probe on the finger at the time of each measurement.

The following experiment determines how the different sensors and sensor types in the fingertip, namely normal force and shear force sensors, respond to a fixed normal force applied at varying locations along the fingertip. The linear table is used to apply a normal force to the finger using the probe on the force/torque sensor. As soon as 2 N of normal force are reached, a measurement of the finger’s sensors is taken. The probe is then lifted again and moved by 0.25 mm along the long axis of the fingertip. The probe is lowered again to apply force and read the resulting sensor outputs. This process is repeated incrementally, starting from the proximal end of the sensorised surface of the fingertip and ending at the distal end. Measurements were taken at an interval of 30 s to limit the influence of hysteresis on the experiment results. The result for the ring finger can be seen in Figure 15.

As can be seen, even a small probe of 5.3 mm could be detected almost everywhere along the fingertip. Only between 16 mm and 18 mm the probe remained hard to detect. Normally, the sensor response should be highest above the sensor itself, so in the case of the barometer-based sensors at the position of the blue pad and in case of the Hall effect based sensors around the golden magnet. As can be seen in the plot this was not the case. The spatial shift in sensor response can be explained by the uneven surface of the fingertip, which can be seen in Figure 4. Since the surface was not even, not all parts of the probe made contact with the finger at all positions. At the curved parts the contact area was smaller and more to the edge of the probe. This in turn shifted the positions of the signals perceived by the sensors.

Together with the observations from Section 3.4, it can be concluded that the spatial resolution of the finger should suffice for use cases concerned with grasping and lifting objects of daily life, while for fine-grained manipulation tasks a higher sensor density can be desirable.

## 4. Discussion and Future Work

In this paper we introduced a concept and implementation of complete scalable robotic fingers with a sophisticated multi-modal sensor system. The fingers are modelled using a skeleton-based parametric model that allows adaptation of all relevant finger dimensions. The embedded electronics are based on readily available sensors and rely on standard design and production techniques. Different sensor modalities have been included in the finger, namely normal and shear force sensors, a distance sensor, an accelerometer as well as joint angle encoders. In addition, each sensor chip includes a temperature sensing element. The sensor system is realised as a number of interchangeable modules that reflect the scalability of the model and allow easy adaptation of the sensor suit to different applications and finger sizes. All tactile sensors are encased in soft silicone while cables and other sensors are encapsulated in the finger itself to increase mechanical robustness. Conceptually, the sensor system is not limited to the presented sensors but can be completely exchanged with any sensor(s) that interface to an electronic bus.

In the experiments presented, we evaluated the tactile sensors, allowing for an informed comparison of two promising tactile sensing methods from literature and show how the detection of distinct events during grasping can benefit from a multi-modal sensor setup. The experiments regarding normal (see Section 3.1) and shear force measurements (see Section 3.2) for the tactile sensors have shown that these are susceptible to hysteresis induced by the silicone. Evaluation of multiple sensors in different fingers shows that this hysteresis, as well as the magnitude of response to forces, is similar for all sensors of each type, indicating that the influence of different shapes of the fingers is minor. The large range of tested sensors also shows that the production process is reliable, as well as repeatable. The density of sensors in the finger is sufficient for the location of the point of contact with an object without larger blind spots (see Section 3.5). Detection of distinct events during grasping and manipulation is not only dependent on tactile sensors but can be realised through sensor fusion of all available sensor data from distance sensors, accelerometers and joint angle encoders (see Section 3.4). The accelerometers have also proven to be a valuable tool for gross slip detection despite being damped by soft material.

In the future we will integrate the presented fingers into our ongoing work on hand prostheses, as well as our humanoid robotic hand development. This will give us the opportunity to further test the robustness of the proposed design as well as validate the usefulness of all parts of the multi-modal sensor setup.

Integration of the fingers into an artificial hand will also make it possible to evaluate different sensor fusion approaches to extract semantic information from the high dimensional sensor information of four fingers. The intention is to utilise the generated information in a similar way to the human, where individual events during grasping like making or breaking contact, lifting and slip seem to define sub-goals during the grasping process [1]. Detection of such events allows breaking down and controlling the grasping process in small steps.

Further experiments are planned regarding the examination of the ability for incipient slip detection based on the accelerometer signals and potential changes of the mounting position of this sensor will be considered.

The addition of further electronics and sensing modalities will also be considered, taking advantage of the modularity of the proposed system. The inclusion of regulated heating elements in the fingers would, for example, enable measurement of temperature flux to objects in contact with the fingers using the temperature sensing elements included in the already present sensor chips.

## Figures and Tables

**Table 1:** Dimensions in [mm] of the finger demonstrators.

	Index Finger	Middle Finger	Ring Finger	Little Finger
Finger length	80.64	90.02	83.98	66.17
Proximal phalanx length	34.15	38.15	34.45	27.05
Distal phalanx length	46.49	51.87	49.53	39.12
Proximal phalanx height	17.28	17.28	17.28	17.28
Distal phalanx height	14.4	14.4	14.4	14.4
Proximal phalanx width	18.7	20.13	18.81	16.5
Distal phalanx width	17	18.3	17.1	15
